# Hitting Is Contagious in Baseball: Evidence from Long Hitting Streaks

**DOI:** 10.1371/journal.pone.0051367

**Published:** 2012-12-12

**Authors:** Joel R. Bock, Akhilesh Maewal, David A. Gough

**Affiliations:** 1 Scalaton, La Mesa, California, United States of America; 2 Department of Bioengineering, University of California San Diego, La Jolla, California, United States of America; University of Westminster, United Kingdom

## Abstract

Data analysis is used to test the hypothesis that “hitting is contagious”. A statistical model is described to study the effect of a hot hitter upon his teammates’ batting during a consecutive game hitting streak. Box score data for entire seasons comprising 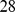 streaks of length 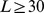 games, including a total 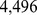 observations were compiled. Treatment and control sample groups (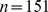) were constructed from core lineups of players on the streaking batter’s team. The percentile method bootstrap was used to calculate 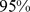 confidence intervals for statistics representing differences in the mean distributions of two batting statistics between groups. Batters in the treatment group (hot streak active) showed statistically significant improvements in hitting performance, as compared against the control. Mean 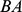 for the treatment group was found to be 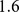 to 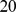 percentage points higher during hot streaks (mean difference increased 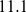 points), while the batting heat index 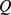 introduced here was observed to increase by 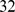 points. For each performance statistic, the null hypothesis was rejected at the 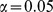 significance level. We conclude that the evidence suggests the potential existence of a “statistical contagion effect”. Psychological mechanisms essential to the empirical results are suggested, as several studies from the scientific literature lend credence to contagious phenomena in sports. Causal inference from these results is difficult, but we suggest and discuss several latent variables that may contribute to the observed results, and offer possible directions for future research.

## Introduction

Baseball folklore has long included the belief that *hitting is contagious*, meaning that when an individual batter’s hit production rate increases over some period (i.e., in a single game, or a consecutive sequence of games), this enhanced facility spreads like an infectious disease to other batters in the lineup. Despite only anecdotal evidence, the idea persists that a hot hitter can transmit this exceptionally difficult mechanical skill to his teammates.

Hot hitting is a transient phenomenon, inherently related to an observation interval. An archetype of unusually hot hitting in baseball is the individual consecutive game batting streak. In particular, streaks of 30 or more games’ duration are rare–only 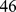 such streaks have been recorded in the Modern Era from 1901 to the present.

The question examined here is whether or not hitting is contagious. The infrequent, long hitting streak provides a model situation to study this question. However, the methods and results of this research have broader implications. If empirical evidence for a contagion effect in baseball were shown to exist, it could provide insights into the psychology of motivation in other team sports, and perhaps more generally into the dynamics of propagation of positive behaviors in sociological, organizational management or economic studies.

### Previous Work

#### Streakiness in sports

Are sports streaks real phenomena, or merely views of random sequences of events misinterpreted by a desire to detect temporal patterns? The “hot hand” has been a bountiful topic for sports-related statistical research.

Streakiness has been studied in connection to many different sports. In a 2006 review, Bar-Eli *et al.* [1] surveyed a large number of studies providing both support and non-support for the belief that “success breeds success and failure breeds failure” in diverse sports. They concluded that most of the empirical research on hot hand effects supported an earlier conclusion by Gilovich *et al.* [2], namely that the probability of a successful shot in basketball was independent of the outcomes on previous shots. Hoewever, simulation studies in different sports [1] suggested that rates of success are non-stationary over time, providing evidence in favor of the hot hand.

Subsequent to the Bar-Eli review [1], reports have appeared in the literature that quantify possible hot hand effects in several sports. Raab and co-workers [3] found evidence for streakiness in some volleyball players’ hit-and-miss patterns; scoring a point made a player more likely to score another in future chances. Moreover, when players were “on a roll” this was detected by their teammates, who were found more likely to pass the ball to the streaking player. In cricket, Ribeiro *et al.* [4] found long-term memory effects by analysis of event-wise scoring in over 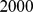 matches. They concluded that a hot hand phenomena exists in cricket, and that this diffusion-like process may unfold over a very long temporal scale. Recently, Yaari and Eisenmann [5] analyzed a large dataset of sequential success/failure rates on free throw attempts documented in NBA basketball. They reported evidence for a hot hand effect, as the probability of success on a free throw improved by 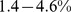 on the second attempt, conditioned on the fact that the first attempt was successful. This was interpreted as strong evidence for a hot hand effect in free throws by NBA players [5].

Baseball hitting streaks were studied by Albright [6], where runs tests and logistic regression models were used to evaluate the existence of batting streakiness. Four years’ Major League batting results were analyzed; the author concluded that batting performance is better explained by a model of randomness, as opposed to objective evidence in support of prevalence in streaky hitting. Albert [7] proposed a *consistent-p* model assuming that for each at-bat during the 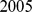 Major League season, the probability of that player successfully getting a hit was constant; it was further assumed that outcomes for different at-bats were independent. Using various metrics, players were evaluated and ranked by streakiness with respect to hits/outs, strikeouts, and home runs. The model in [7] was shown to explain most of the intra-seasonal variation in streaky hitting. Quintana *et al.* [8] developed Bayesian models to investigate sequential hitting success (incorporating hits, walks and sacrifices) spanning four complete seasons. They did not find evidence that streakiness of individual players persisted from season-to-season. The most important covariates with situational hitting success were found to include: (1) the number of outs at time of plate appearance; (2) the number of runners on base; and (3) game location at batter’s home field; and (4) the earned run average (ERA) of the opposing pitcher [8].

#### Previous studies of statistical contagion

These investigations applied statistical methods to analyze an individual streaky player, or that of aggregate behavior. The identification of contagion effects requires consideration of a streak’s effect as it spreads to teammates. The metaphor of contagion suggests utilizing analytical methods developed in epidemiology as a framework for scientific investigation.

The first Surgeon General’s report in 1964 [9] established the U.S. government’s position that scientific evidence suggested a causal relationship between cigarette smoking and lung cancer. This report advanced a number of criteria to identify causal relationships between variables, including: consistency (reproducibility over time and location), strength of association, specificity of association, temporality (cause precedes effect), and coherence (concurrence of collective evidence). These criteria for evaluation of either an association or true causative effect between environmental feature *A* and a consequent event *B* were reviewed extensively by Hill [10]. In a recent study of epidemiological literature, Parascandola *et al.* [11] found that strength and coherence were most often used in practice to establish causal inference; consistency was moderately used, and temporality and specificity were not applied at all in some cases. This suggests that over time, scientific approaches to causal inference in an epidemiological setting have a reduced emphasis on temporality as an essential prerequisite to statistical demonstration of causality.

Contagious feelings in social groups have been widely studied. Hatfield *et al.* [12] described the process of emotional contagion as one in which “…people nonconsciously and automatically mimic their companions’ fleeting expressions of emotion…people can and do `feel themselves into’ the emotional landscapes inhabited by their partners.” They concluded that from moment-to-moment, people tend to “catch” others’ emotions, and cite literature from a wide spectrum of fields in support of this conclusion.

Barsade [13] conducted experiments on different aspects of mood propagation amongst groups, and concluded that emotional contagion does exist within groups. Positive emotional contagion was correlated with better cooperation, reduced conflict, and enhancements in perceived task performance. In the sporting world, Moll and investigators [14] uncovered association between team celebrations after successful soccer penalty kicks and the ultimate outcome of a penalty shootout. This was attributed to the spread of a positive attitude throughout the team during the sequence of shots. The opposite effect was seen on the opposing team–after a successful kick, the opponents’ next try was more likely to result in a miss if certain behaviors were exhibited by the previous, successful kicker. On the cricket pitch, Totterdell [15] found evidence for what he called “mood linkage” on sports teams, which contributed to correlation between a positive overall team mood and a players’ mood as well as self-appraisal of his performance. Experiments reported by Lee *et al.* [16] showed that golfers who believed they were using a club previously used by a professional golfer realized improved putting performance. Specifically, subjects perceived that the golf cup itself had increased in physical dimension. The authors [16] assigned this to a *positive contagion* effect from using the pro golfer’s equipment.

There may be a neurobiological mechanism explaining such observations, suggesting a connection between observation of sports behavior and its propagation to observers of that action.

Rizzolati *et al.* [17] reviewed a large number of “mirror neuron”-related studies, which show that simple conceptualization of limb movements produces activity in the same brain areas that are involved in producing the actual movements themselves. The ventral premotor cortex has both cognitive (space perception, action understanding and imitation) and motor functions, the latter of which transform object properties into hand actions, and spatial locations into head and arm actions. Cross and co-workers [18] found experimental evidence for a common neural substrate for both observational and physical learning. The authors concluded that it is possible to achieve new action learning from passive observation.

Gray and Beilock [19] reported on a simulation experiment on the psychological mechanism of “action induction”, whereby observation of the actions of a hot hitter in turn improve the batting performance of the observer. While real game data was not part of this study, action induction was proposed as a sensorimotor explanation for the belief that hitting is contagious.

#### Homophily or social influence

It is important to recognize the potential impact of unobserved variables before declaring that a causal relationship is fundamental in any contagious hitting effect. The work of Shalizi and Thomas [20] suggests that in general, it is virtually impossible to distinguish between influence and “latent homophily” in social networks. Under social influence (or contagion), The diffusion of behaviors corresponds to the idea of contagion, as behaviors change in order to be more similar to others in the group. Homophily, on the other hand, suggests the formation of social connections due to pre-existing similar attributes among individuals. As applied to the present study, important analogic incongruities are present. Unlike online social networks, in the baseball setting, the population on each team is small and fixed; associations are controlled by the manager who constructs the lineup. Baseball teams don’t self-organize; baseball ownership draft or trade for players based on economics and skillset requirements at different positions. The effect of location in the batting order is the closest conceptual analog to a linkage change in a dynamic social network; however, this change is controlled by the manager, not the players, precluding the application of statistical tests to discriminate homophily versus influence (for example, see [21] and [22]). Despite these discrepancies in analogy, we recognize that unobserved covariates may be important in shaping the observed results. Our discussion considers a number of possible latent factors, including batting order position, opposing pitching quality, latency in streak recognition, and overall team skill level.

#### Contribution of this research

The purpose of this study was to examine the hypothesis that “hitting is contagious” in baseball. A retrospective analysis was undertaken on box scores from 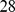 entire seasons during which long hitting streaks were accomplished. An hypothesis test was formulated to investigate the treatment effect of the hot hitter on his teammates. Results obtained from the aggregated sample (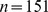) suggest the demonstration of a “statistical contagion effect”. This work offers a contribution to the literature where investigators found evidence for positive contagion in other sports, such as soccer [14], cricket [15] and golf [16].

There appear to exist no previous empirical studies within the framework of confirmatory data analysis to quantify the spread of hot hitting in baseball. This approach appears to be novel, and could be applied to studies of performance enhancement in other team sports, or extended to sociological, organizational and economic investigations.

## Methods

### Population and Sample

The implied population for the present study is the set of all Major League Baseball (MLB) players active since 1945. The experimental sample culled from this population comprises players who were teammates of one of the players achieving a consecutive game batting streak of length 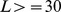 games spanning one or two seasons. In order to perform meaningful inference, we select only those players within this sample whose average number of at-bats per game exceeded a threshold value 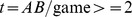. We refer to this subsample as the “core lineup” for each hitting streak under consideration. The reader will note that our specific interest is on at-bats versus plate appearances, the latter of which may include walks, hit-by-pitch, sacrifices, or other outcomes not indicative of hot hitting.

We partition the sample into two groups: a treatment group 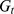 and a control group 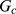. The same individuals are observed both during the hot hitter’s streak (treatment) and when the streak is not active (control) in the context of a season. Inclusion of the same individuals within each group in the sample design reduces the potential for selection bias [23].

A sufficiently randomized sample is achieved by collecting data from both Major Leagues, over many seasons, thereby mitigating undue influence of potential sample biasing factors including: (a) the raised mound and expanded strike zone (ca. 1963–1968) which tended to favor pitchers; (b) the “steroid era” (approx. 1988–2010) which favored batters using performance-enhancing substances; and (c) subjective differences in strike zones between the two leagues favoring pitchers (National League: low zone) and batters (American League: high zone), respectively [24].

Finally, our sample streaks postdate the Lively Ball Era (1920–1945) where batters were at a distinct advantage.

### Box Scores Database

The box score data analyzed in the present study were obtained from the online resource Baseball-Reference.com (http://www.baseball-reference.com). Our analysis centers on 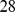 long streaks in the post-WWII era as listed in Table 1. Season-long data for the batting streaks subject to investigation represented in total 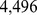 observations.

**Table 1:** Hitting streaks of length 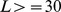 games since 1945.

ID	Player	Team	Year	Finish	*L*	*#CL*
1	T. Holmes	BOS Braves	1945	6/8	37	4
2	D. DiMaggio	BOS Red Sox	1949	2/8	34	7
3	S. Musial	STL Cardinals	1950	5/8	30	6
4	V. Pinson	CIN Reds	1965(6)^1^	4/10	31	6
5	W. Davis	LA Dodgers	1969	4/6	31	6
6	R. Carty	ATL Braves	1970	5/6	31	5
7	R. LeFlore	DET Tigers	1975(6)^1^	6/6	31	5
8	P. Rose	CIN Reds	1978	2/6	44	5
9	G. Brett	KC Royals	1980	1/7	30	9
10	K. Landreaux	MIN Twins	1980	3/7	31	7
11	B. Santiago	SD Padres	1987	6/6	34	6
12	P. Molitor	MIL Brewers	1987	3/7	39	6
13	J. Walton	CHI Cubs	1989	1/6	30	5
14	H. Morris	CIN Reds	1996(7)^1^	3/5	32	4
15	N. Garciaparra	BOS Red Sox	1997	4/5	30	7
16	S. Alomar, Jr.	CLE Indians	1997	1/5	30	7
17	E. Davis	CIN Reds	1998	4/6	30	6
18	L. Gonzalez	ARI Diamondbacks	1999	1/5	30	5
19	V. Guerrero	MON Expos	1999	4/5	31	4
20	L. Castillo	FLA Marlins	2002	4/5	35	5
21	A. Pujols	STL Cardinals	2003	3/6	30	4
22	J. Rollins	PHI Phillies	2005(6)^1^	2/5	38	6
23	C. Utley	PHI Phillies	2006	2/5	35	4
24	W. Taveras	HOU Astros	2006	2/6	30	7
25	M. Alou	NY Mets	2007	2/5	30	5
26	R. Zimmerman	WSH Nationals	2009	5/5	30	3
27	A. Ethier	LA Dodgers	2011	3/5	30	4
28	D. Uggla	ATL Braves	2011	2/5	33	4

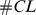
 is the size of the core lineup that in total constitutes the sample population for this study.

1 In streaks spanning two seasons, only the season with the majority of games comprising the streak is considered.

Raw box scores were downloaded manually in comma-separated value (CSV) format. These files were annotated according to the dates of activity of the associated hitting streak; this annotation formed the basis for partitioning the batters into the two sample groups. The aggregate sample sizes for each group were identical (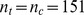, the total of the core lineup column in Table 1).

This database of box score data was subsequently analyzed using the statistical methods that are described in the section on *Analysis*.

### Analysis

#### Hot hitting statistics

How shall we define “hotness”? Our model situation for studying the contagion of hot hitting is the consecutive game hitting streak. By definition, it is the *length* of the streak itself that is the primary distinguishing factor. The batting average, the ratio of number of hits 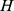 to at-bats 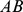,
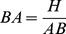
(1)is the most widely understood and fundamental measure of hit production by a player. According to MLB rules, just a single base hit per game (with at least one qualifying at-bat) is enough to perpetuate a consecutive game hitting streak. While virtually unobserved historically, it is possible under the rules to post a low batting average during a long (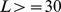) game streak.

By extension, in the putative measurement of hot hitting contagion throughout the dugout, it is possible that the batting average statistic alone may not be a sufficiently sensitive indicator.

Long runs of consecutive games with at least one hit are not realized by the streaking batters’ teammates; otherwise they would constitute noteworthy streaks in and of themselves. However, short bursts of “microstreaks” coincident with the hot batter’s streak are observed and can be quantified.

To assess offensive production by the core lineup players constituting each sample group, we propose a statistic that expresses both microstreak length (run length of consecutive games with 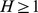) and batting average to express the quality of batting performance. Let us define a batting heat index over the 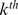 microstreak by the 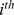 core lineup member as
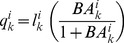
(2)where 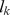 is the run length in games, and 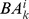 is the player’s batting average for games occurring over this interval. For runs 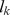 where no hits are produced, the value of 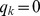, precluding heat accumulation over hitless microstreaks. The core lineup batter realizes many such clusters (total 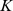) of short-term streaks within the course of the hot hitter’s streak, which lasts for 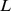 games. The overall heat index for this player is compiled and normalized as



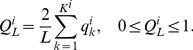
(3)The heat index of Eqn. 3 represents both the persistence and density of hit production by the core lineup player over the interval 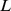.

As an illustration, consider sequences of hits per game as produced by two different hitters. Suppose that for a notional 13 game interval, each player records 4 at-bats per game. The hit totals for each player are, respectively,
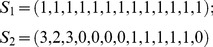
(4)


The first hitter’s 13-game hitting streak yields statistics 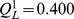, 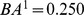; the second hitter’s statistics over this interval are 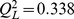, 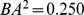. The batting averages are identical. As measured by 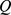, the second player’s ephemeral hotness as compared to the streak hitter can be quantified, although his 2 microstreaks are not extraordinary events.

The statistics 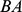 and 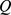 were computed from box score data for the core lineup players comprising each sample group (*treatment* 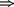 streak active; *control* 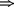 streak inactive).

**Figure 1:**
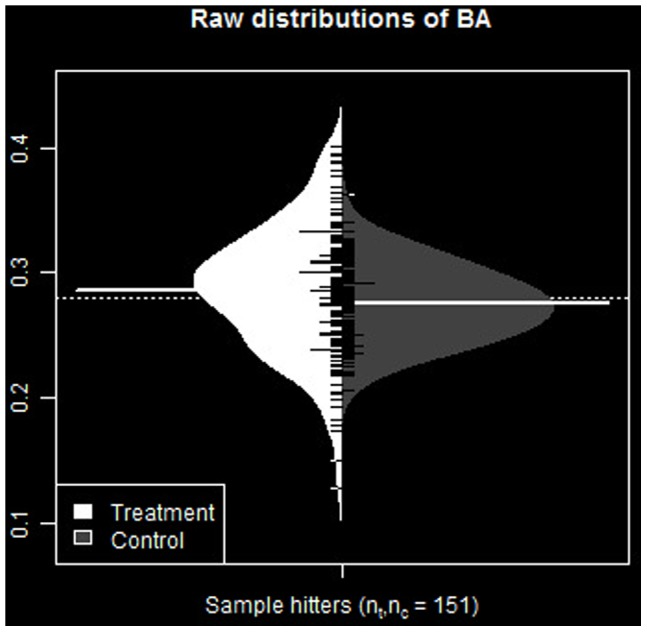
Distribution of *BA* statistic for sample groups. Original sample data representing the population from which resampled statistics are drawn, and ultimately used to construct bootstrap distributions and confidence intervals.

**Figure 2:**
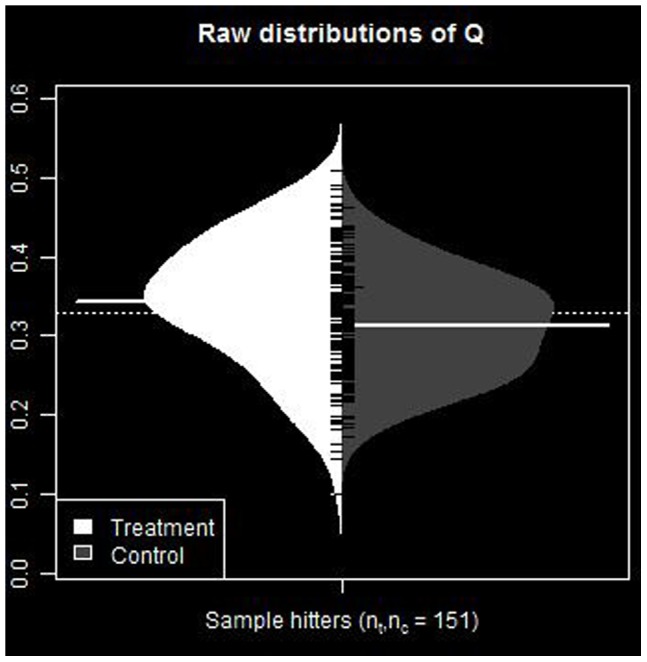
Distribution of *Q* statistic for sample groups. Original sample data representing the population from which resampled statistics are drawn, and ultimately used to construct bootstrap distributions and confidence intervals.

Comparative distributions of raw numerical values for these statistics for the two groups are presented in Figures 1 and 2. These figures display, side-by-side, distributions of the hot hitting statistics between groups. These are the original sample data representing the population from which resampled statistics are drawn, and ultimately used to construct bootstrap distributions and confidence intervals. They provide a visual description the relative distributions of values of the statistics between groups.

In the next section, we describe hypothesis tests applied to the distributional differences between groups based on this sampling of the population.

#### Hypothesis tests

Let us define hitting statistics for the differences in group wise means of the distributions of batting average and batting heat index:
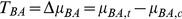
(5)

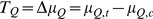
(6)


The null hypothesis 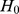 assumed no difference between groups. In this investigation, the interpretation of the null hypothesis was that hitting is not contagious. The alternative hypothesis 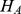 was that hitting *is* contagious. Symbolically, these hypotheses are written
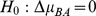


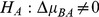
(7)

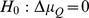


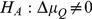
(8)for the batting average and heat index tests, respectively.

#### Bootstrap confidence intervals

The null hypothesis was tested using bootstrap resampling to calculate nonparametric confidence intervals (CIs) around the statistics for the differences in group wise means, 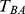 (Eqn. 5) and 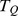 (Eqn. 6).

Efron introduced bootstrap methods [25], which have been shown to be useful for a large variety of statistical estimation problems. Here, bootstrapping is used to esimate the value of a statistic describing a population by repeated resampling of the original sample representing the population, computing the statistic for each replicate, and finally constructing a “bootstrap distribution”–an approximation of the shape, variance and bias of the sampling distribution of the sample statistic.

In principle the distribution of nearly any real-valued statistic may be examined using the bootstrap procedure. The statistics 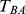 and 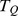 express the difference in means of distributions for the treatment and control groups. We used the percentile method [26] with 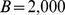 bootstrap replicates to estimate empirical distributions of the resampled statistics; the 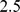 and 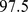 percentiles of these distributions were taken to constitute the limits of the 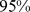 CIs. Analysis of bootstrap differences in means between the sample groups was carried out using the simpleboot package [27] within the *R* statistical computing environment [28].

Locations of the values of the statistics observed from the original sample were compared to these CIs in order to infer the presence or absence of a significant (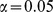)“treatment effect” of streaky hitters upon their teammates.

Our two-sample bootstrap procedure is described by the following steps [29]:

1. Draw distinct resamples of size 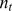, 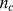 with replacement from treatment and control samples, respectively;
2. Compute statistics (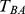, 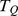) from these resamples;
3. Repeat 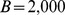 times;
4. For each statistic, construct sampling distributions and estimate 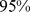 confidence intervals.

## Results

**Figure 3:**
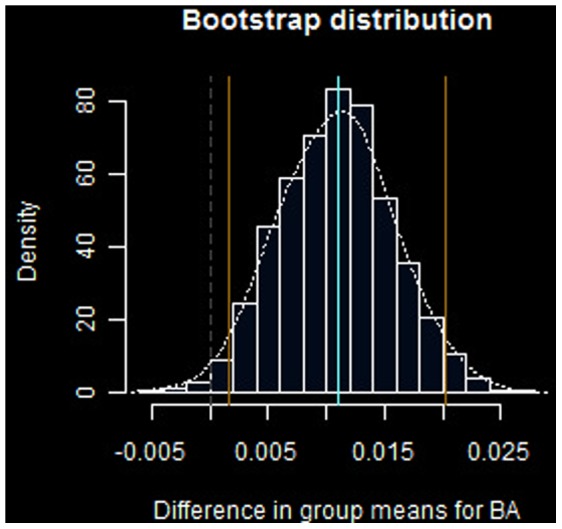
Bootstrap distribution for for difference in group wise means, *T_BA_*. Blue lines denote 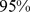 confidence interval 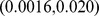 around a mean difference 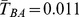, or 11 percentage points. Sample size 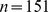, 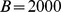 replicates.

**Figure 4:**
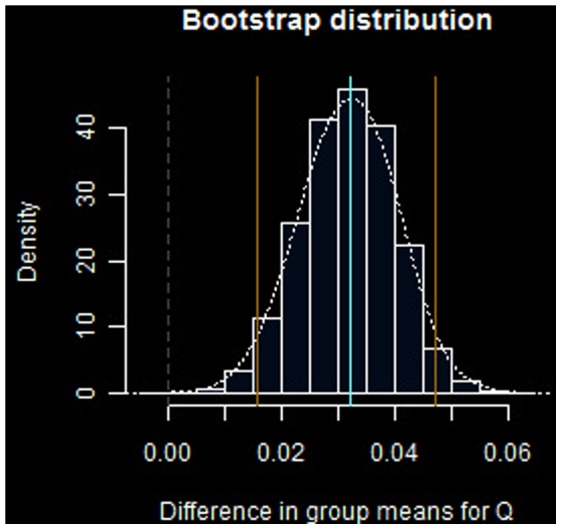
Bootstrap distribution for difference in group wise means, *T_Q_*. Blue lines denote 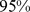 confidence interval 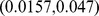 around mean difference 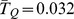. Sample size 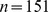, 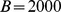 replicates.

The main results of the experiments are summarized in Figures 3 and 4.

The bootstrap distribution for batting average difference between groups 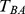 (Eqn. 5) is displayed in Fig. 3. The estimated limits of the 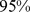 confidence interval for this statistic are 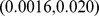 around a mean difference of 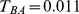. CI limits are shown in blue in the figure. The dashed line marks the location of the mean of the distribution under the null hypothesis.

For the batting “heat index” metric, bootstrap distribution results for differences between the treatment groups (Eqn. 6) appear in Fig. 4. The 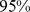 CI calculated for 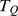 is 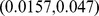, centered about a mean difference 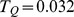.

## Discussion

Our results show that for the batting average, the null hypothesis of “no difference between groups” is rejected at the 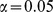 level. We submit that this suggests the existence of a *statistical contagion effect* for hot hitting. The aggregate 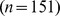 *BA* was seen to increase by 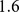 to 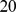 points (average of 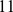 percentage points) for the treatment group during a teammate’s hitting streak.

The null hypothesis is also rejected for the heat index statistic *Q*. This provides additional evidence in support of the conjectured alternative hypothesis, according to the methods and assumptions of this study. The mean value of *Q* improved by 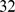 points relative to the control group. The *Q* effect is apparently more pronounced than that seen for *BA*, as indicated by the location of the distributional mean under the null hypothesis in Fig. 4 relative to the confidence interval.

We reject the null hypothesis. However, this does not prove the truth of the alternative hypothesis; that is, we cannot claim to have demonstrated a direct causal relationship between a hot hitter’s streak and improved hitting performance of his team.

The observed results may be generated by any number of latent factors. Some of these are discussed below.

### Batting Order Position

A player’s position in the batting order relative to that of the streak hitter might correlate with the quality of pitching he experiences, thereby contributing to the observed effect. To study this variable, mean values for offensive statistics posted by players in each sample group were associated with their average relative lineup position for the games under consideration.

**Figure 5:**
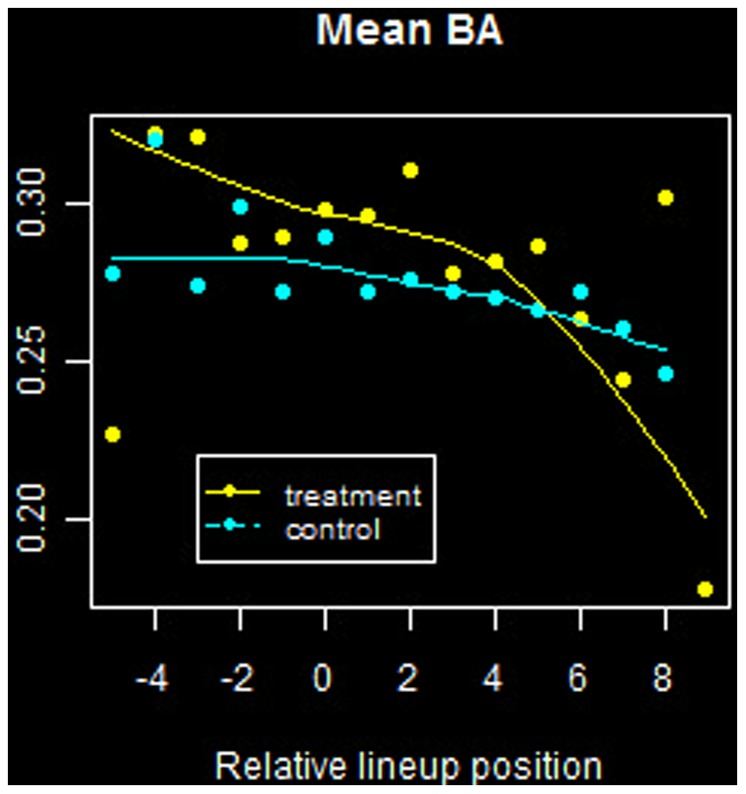
Mean *BA* versus relative lineup position. Offensive statistics for players in each sample group as a function of batting order. Negative values along the abscissa correspond to batting before the streak hitter.

**Figure 6:**
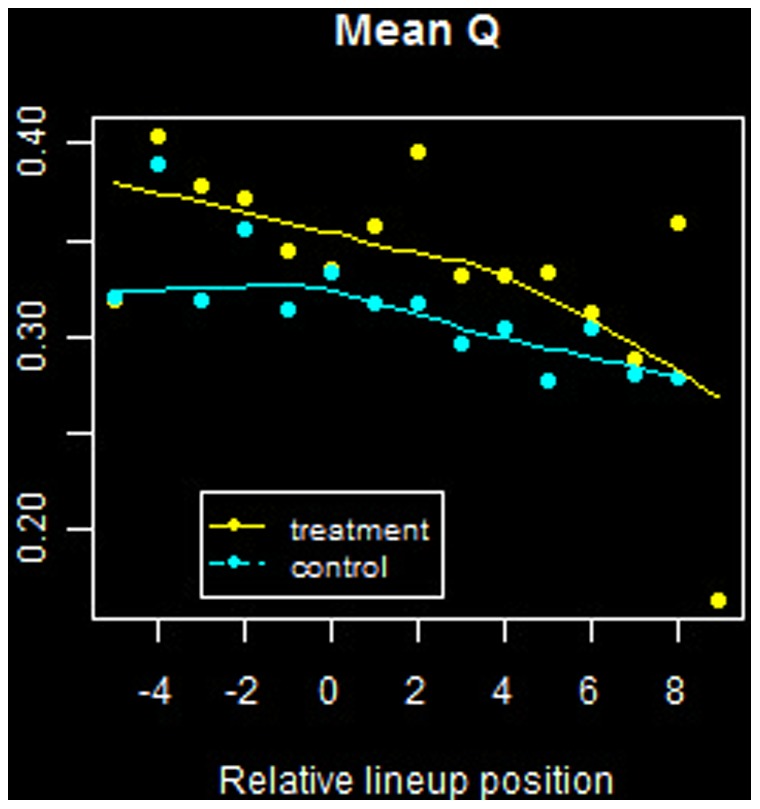
Mean *Q* versus relative lineup position. Offensive statistics for players in each sample group as a function of batting order. Negative values along the abscissa correspond to batting before the streak hitter.

The results are summarized in Figures 5 and 6 for 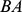 and 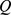, respectively. In each figure, due to large variance in the data, LOESS curves with a first degree polynomial have been overlayed as a visual aid.

Consider Figure 5. It is apparent that batting averages tend to be higher the earlier in the lineup that a player is positioned (negative values along the abscissa correspond to batting before the streak hitter). The general decrease in 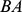 from top to bottom of the order is observed for both treatment and control groups; this is as expected, as managers devise their lineups to increase plate opportunities for more productive hitters, in an average sense.

Similar trends are seen in terms of the heat index 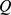, shown in Figure 6. For both groups, the average value of this indicator declines as a function of position in the batting order, and this may be again attributed as per design by the team manager.

We conclude that batting order position is not an explanatory factor in the hitting contagion phenomenon.

### Streak Recognition Delay

One of the criteria for causal infererence that was identified in the Surgeon General’s report [9] was temporality, or establishing that the cause precedes the effect. Hill noted [10] that this criterion is important in the spread of diseases which are slow to develop. Parascandola *et al.* [11] later found that the scientific literature often ignored the criterion of temporality when making the case for epidemiological causality.

In terms of the present study, a reasonable question is whether the streaking batter’s teammates notice a change in behavior in the early games of a nascent hot streak. We wondered if the contagion effect would manifest as statistical improvement after some period of latency following the official onset of the hitting streak.

The procedure followed to partition the sample groups in this investigation assumed that the treatment group immediately recognizes that their teammate is “hot”. In practice, a hot batting streak in baseball usually eludes diffuse media attention before having progressed for at least 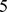 or 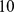 games.

To simulate this situation, we carried out the bootstrap resampling procedure and computed 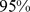 confidence intervals for the distributions of the differences in means between groups, as detailed in the Section *Hypothesis tests*. For this analysis, the assignment of the “treatment” effect was delayed by several games (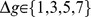) after the streak actually began. Because termination of a long streak is obvious, in this model the treatment effect ends in coincidence with the end of the streak.

The results of this analysis are compiled in Table 2. Mean values of the bootstrapped group wise differences in means (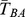 and 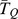) are listed in the table, alongside the corresponding decision to accept or reject the null hypothesis 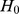 which states that that hitting is not contagious. For reference, the results for a zero game recognition lag (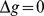) are included in the table in the first data row.

**Table 2:** Effect of streak recognition delay.

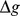	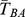	Reject *H*_0_?	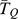	Reject *H*_0_?
**0**	**0.011**	**Y**	**0.032**	**Y**
1	0.009	N	0.025	Y
3	0.005	N	0.018	Y
5	0.005	N	0.018	Y
7	0.000	N	0.011	N

Mean values of bootstrapped group wise mean differences for various streak recognition delays 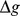.

**Table 3:** Batting statistics for the streak hitters of this study.

ID	Player	Year	Δ*BA*	Δ*Q*
1	T. Holmes	1945	0.0898	0.1491
2	D. DiMaggio	1949	0.0582	0.0516
3	S. Musial	1950	0.0543	0.1432
4	V. Pinson	1965	0.0803	0.0972
5	W. Davis	1969	0.1654	0.3488
6	R. Carty	1970	0.1116	0.2635
7	R. LeFlore	1976	0.0975	0.1860
8	P. Rose	1978	0.1168	0.1930
9	G. Brett	1980	0.1063	0.3332
10	K. Landreaux	1980	0.1497	0.2982
11	B. Santiago	1987	0.0560	0.1456
12	P. Molitor	1987	0.0957	0.3035
13	J. Walton	1989	0.0639	0.2546
14	H. Morris	1996	0.0729	0.1558
15	N. Garciaparra	1997	0.0975	0.1470
16	S. Alomar, Jr.	1997	0.1329	0.2392
17	E. Davis	1998	0.1019	0.2651
18	L. Gonzalez	1999	0.0848	0.0684
19	V. Guerrero	1999	0.0877	0.1541
20	L. Castillo	2002	0.1303	0.1642
21	A. Pujols	2003	0.0389	0.0221
22	J. Rollins	2005	0.1172	0.2016
23	C. Utley	2006	0.1260	0.2107
24	W. Taveras	2006	0.0977	0.2302
25	M. Alou	2007	0.1015	0.3384
26	R. Zimmerman	2009	0.1145	0.1831
27	A. Ethier	2011	0.1223	0.2675
28	D. Uggla	2011	0.1838	0.2547

Values are expressed as differences in/out of their streaks (see Table 1). These hitters were excluded from the sample population.

For the 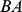 statistic, a recognition time delay of any number of games results in failure to reject 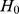 at the 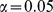 significance level. This is true even for a single game delay 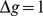, as the distribution represented in Figure 3 shifts to the left enough such that the 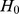 mean falls within the bootstrapped confidence interval. The 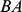 contagion effect disappears when there is a delay in identification of the streak on the part of the team.

A different result is seen for the heat index 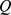 within the delayed recognition model. In terms of this statistic, the contagious effect persists up to a lag of 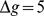 games. One interpretation of this observation is that when recognition latency is present, the heat index is more sensitive at detecting the spread of hot hitting throughout the lineup (as compared to 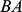). The association between cause and presumed effect is stronger according to this metric.

### Opposing Pitching Quality

The quality of opposing pitching facing the sample groups is another variable potentially influencing the results of this study. Although unlikely over the course of a 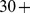 game streak, it is possible that collective pitching performance faced by the treatment sample is somehow inferior in contrast to the control.

Analysis of the isolated importance of “pitching quality” would be complicated. One difficulty lies in the task of separating pitching from hitting–the two factors are clearly not independent of one another. As baseball philosopher Casey Stengel once remarked, “*Good pitching will always stop good hitting and vice-versa*” (http://www.baseball-almanac.com/quotes/quosteng.shtml).

Including pitching quality as an essential variable was deemed beyond the scope of the present investigation. Future research into the role of pitching to suppress hot hitting may be informed by the following notes.

1. A detailed perspective could be obtained by expanding the present analysis to consider the individual batter versus pitcher matchups for each at-bat. This type of information is available from at least two readily accessible resources (www.baseball-reference.com, and www.retrosheet.org). The constitution of the core lineups would probably be changed, because of the requirement for an average, minimum number of at-bats as imposed here. This might reduce the sample size considerably.
2. Owing to the fact that a large number of different pitchers are seen by a team over course of a season, an important design parameter would be to establish rational criteria for a requisite number of pitchers’ innings. Managers can always bring in fresh arms from the bullpen when necessary.
3. A more general view might be accomplished by the formulation of a composite Earned Run Average (ERA) realized against both hitting groups. However, the ERA statistic includes many means to reach base and ultimately score (walks, hit batters, sacrifices) not accounted for using the present hot hitting statistics; this would still be problematic for drawing inference from pitching as a factor. One of many other quality statistics that might be considered is the so-called “Pitcher Dominance Factor” proposed by former MLB pitcher Curt Schilling [30].
4. The conditional dependence of hitting and pitching could be partially mitigated through the implementation of a Nave Bayes computational approach, for example, as discussed in Duda and Hart [31].

### Overall Team Skill

It is conceivable that the observed hot hitting results might be due to a greater concentration of skilled players on certain teams relative to the competition. This relates to the dilemma of discerning homophily from influence [20]; better hitters might already have coalesced onto certain teams, and the contagious hitting is due to this structural grouping as opposed to a diffusive effect. If some teams were generally more highly skilled, we would expect that teams with players achieving long hitting streaks would dominate, and routinely finish at or near the top of their respective divisions at the conclusion of the streak seasons.

As an indicator of overall team skill, we considered the final standings for each team in the present study. These standings are listed in the *Finish* column of Table 1. The first number is the numeric standing (lower numbers mean a higher finish), and total number of teams in that division is shown after the backslash.

The mean finishing position for the streak teams was 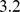 out of 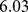 teams/division, or slightly worse than the middle of the pack in the division. All other factors being equal, this implies that these teams did not possess uncommonly talented players as compared to their competition. Good, mediocre and bad teams experience hot hitting streaks. Therefore, overall team skill level is ruled out as an explanation for the observed contagion effect.

### Mechanisms of Contagion

We observed evidence of a statistical contagion effect. The preceding discussion considered a number of possible latent external covariates that might account for our observed results. If hitting contagion does have a concrete basis, it is likely be motivated internally; some neurobiological or psychological mechanisms then would translate the identification and observation of hot hitting by the streak hitter into an improvement in hitting performance by the observer.

We briefly point to four distinct studies from the scientific literature that attempt to explain mechanisms of the transduction of observation into performance by the observer.

In a study particulary germane to our investigation, Gray and Beilock [19] advanced the idea of the mechanism of “action induction” to explain hot hitting contagious effect. In action induction, observers of the hot hitter tended to imitate performance of others’ actions that were recently observed. An experimental study by Cross *et al*. [18] identified a neurological pathway that associates new action learning from passive observation; the implications for the present work are immediate and obvious. Rizzolati *et al.* [17] summarize many studies on mirror neurons that fire in the mind of an observer watching others perform physical activities; these same neurons are associated with limb movements used in actual performance of this activity. Finally, the work of Lee and co-workers [16] demonstrated a positive contagion effect on golfers who erroneously thought they were using putters belonging to highly skilled golf professionals.

### Concluding Remarks

A fascination with statistics is one of the hallmarks of fans of American baseball. Several interesting extensions to the present work can be envisioned.

Other statistics indicative of hot hitting might be used to augment those used here (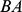 and the heat index 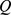). For example, the on-base plus slugging percentage (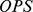) might be incorporated to provide information on different aspects of offensive output (walks and power hitting) by core lineup players during a hot streak.

Supplemental studies might investigate the time course of a hot hitting “epidemic” as a streak extends in duration, perhaps carrying the metaphor forward by employing analytical methods from epidemiology to the extent that additional insight may be achieved into the mechanisms of transduction.

Finally, it is of interest to note how the streaking batters themselves performed using our statistical indicators during their long hitting streaks. In Table 3, we present data summarizing differences 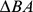 and 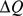 for the model streaks considered in the current investigation. These batters were not included in the sample groups subjected to hypothesis testing.

### Supporting Information

The hitting streak box scores data are provided in Dataset S1.

## Supporting Information

Dataset S1Box score data used in this research. The archive file contains one folder for each of the 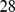 batting streaks, named according to streak batter, year and length of the streak. Individual game box score filenames indicate date of occurrence relative to the streak (beginning with characters “B” for before streak, “A” for after streak). Raw data were obtained from www.baseball-reference.com.(ZIP)
