# Correction: Hitting Is Contagious in Baseball: Evidence from Long Hitting Streaks

**DOI:** 10.1371/annotation/5c16bd9b-5a6b-4d29-bafa-4d0a92552db2

**Published:** 2013-08-16

**Authors:** Joel R. Bock, Akhilesh Maewal, David A. Gough

The figures in the article were corrupted. The correct figures are listed below.

Figure 1: 

**Figure:**
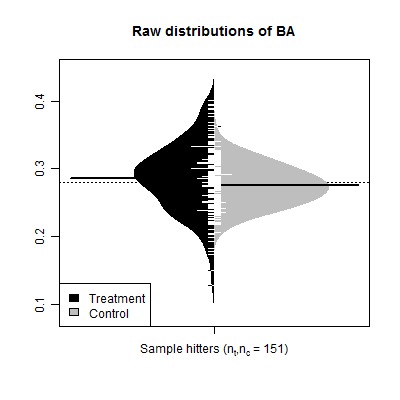


[^]

Figure 2: 

**Figure:**
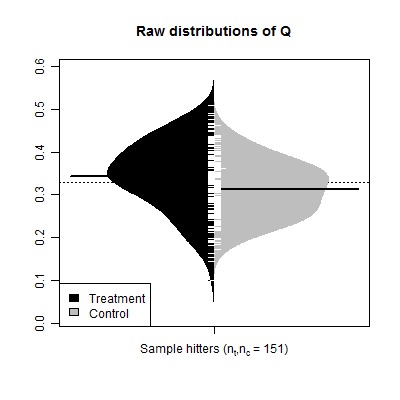


[^]

Figure 3: 

**Figure:**
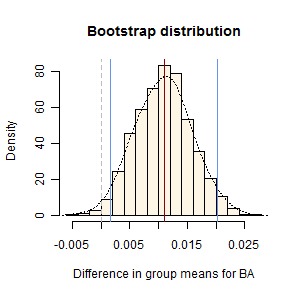


[^]

Figure 4: 

**Figure:**
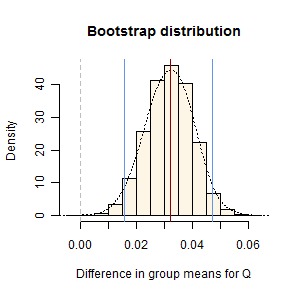


[^]

Figure 5: 

**Figure:**
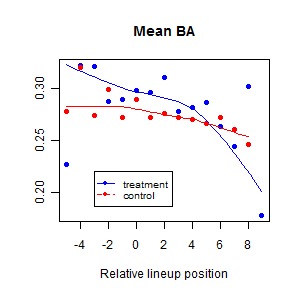


[^]

Figure 6: 

**Figure:**
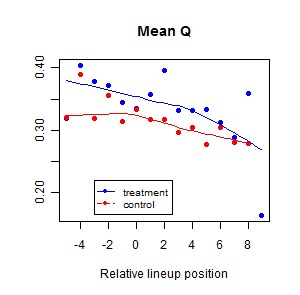


[^] 

